# Transcript accumulation rates in the early *Caenorhabditis elegans* embryo

**DOI:** 10.1126/sciadv.adi1270

**Published:** 2023-08-23

**Authors:** Priya Sivaramakrishnan, Cameron Watkins, John Isaac Murray

**Affiliations:** Department of Genetics, Perelman School of Medicine, University of Pennsylvania, Philadelphia, PA 19104.

## Abstract

Dynamic transcriptional changes are widespread in rapidly dividing developing embryos when cell fate decisions are made quickly. The *Caenorhabditis elegans* embryo overcomes these constraints partly through the rapid production of high levels of transcription factor mRNAs. Transcript accumulation rates for some developmental genes are known at single-cell resolution, but genome-scale measurements are lacking. We estimate zygotic mRNA accumulation rates from single-cell RNA sequencing data calibrated with single-molecule transcript imaging. Rapid transcription is common in the early *C. elegans* embryo with rates highest soon after zygotic transcription begins. High-rate genes are enriched for recently duplicated cell-fate regulators and share common genomic features. We identify core promoter elements associated with high rate and measure their contributions for two early endomesodermal genes, *ceh-51* and *sdz-31*. Individual motifs modestly affect accumulation rates, suggesting multifactorial control. These results are a step toward estimating absolute transcription kinetics and understanding how transcript dosage drives developmental decisions.

## Teaser

Many embryonic genes are extremely rapidly transcribed, which involves unique genomic characteristics and promotes robustness.

## INTRODUCTION

The embryonic transcriptome is highly dynamic, and zygotic transcription in the right cells, at the right time and at the right levels, is important for robust cell fate decisions during early development. However, absolute transcript accumulation rates (rate of change of RNA copy number) that lead to timely production of transcripts at the appropriate level are rarely explicitly measured at a global scale during development. Quantitative imaging of transcriptional activity during development, performed most extensively in *Drosophila* embryos, has provided detailed measurements of the transcript production rates of some early patterning genes (*1*, *2*). One such study found the accumulation rates of the gap genes *hunchback*, *krüppel*, *knirps*, and *giant* to be fairly similar (33 transcripts/min per nucleus) during the 15-min nuclear cell cycle 13 (*3*). These rates are high, with levels approaching the theoretical steric limit based on the RNA polymerase II (PolII) footprint, which would allow for one PolII every 70 to 80 base pairs (bp) (*4*, *5*). These measurements have been borne out by live imaging of mRNAs, which has provided substantial details on the underlying dynamics of rapid accumulation, including the rates of transcription elongation and the response of cis-regulatory elements to input maternal gradients (*1*, *6*, *7*). However, live transcript imaging often relies on transgenes, missing the native gene context. Furthermore, most fly studies focus on the early embryo, which is syncytial (with nuclei sharing common cytoplasm), and it is less clear whether similarly high accumulation rates are common in cellularized embryos or if high rates extend beyond the well-studied patterning genes. Genome-wide bulk RNA sequencing (RNA-seq) measurements of transcript accumulation on a broad time scale (multiple rounds of cell division) in the *Xenopus* embryo similarly showed that most genes, including transcription factors (TFs), had uniform accumulation rates of ~10^5^ transcripts/hour per whole embryo (*8*). Averaging this accumulation over multiple contributing cells may correspond to a much lower per cell rate, leaving open the question of how rates are controlled in individual cells, and the impact of rate dysregulation on developmental fate decisions remains underexplored.

The *Caenorhabditis elegans* embryo provides an ideal developmental system to answer these questions. It develops with an invariant lineage where division patterns are highly reproducible across embryos, allowing equivalent cells to be compared between individuals (*9*, *10*). This robustness results, in part, from a high level of precision in expression levels of cell fate specification genes, which has been observed for intestinal TFs (*11*, *12*). The intestinal specification GATA factors *end-3* and *end-1* reproducibly accumulate to very high maximum transcript levels (>300 mRNA molecules per cell) in short (15 to 20 min) cell cycles, suggesting that these genes are transcribed at very high rates (*11*, *12*). Proper gut formation requires these transcripts to surpass a threshold level, emphasizing the importance of transcription rate in developmental progression (*11*, *13*).

Single-cell, single-molecule transcript imaging methods have been indispensable for visualizing and measuring transcript changes and quantifying the absolute number of mRNA molecules during development (*14*, *15*). Single-molecule RNA fluorescence in situ hybridization (smFISH) in fixed embryos has highlighted the importance of precise transcript levels for developmental robustness (*3*, *11*, *16*). However, smFISH is not easily scalable for genome-wide interrogation of transcription rates. Studies in cell culture have shown a strong correlation between reads from different single-cell RNA-seq (scRNA-seq) platforms and smFISH transcript counts, alluding to the potential for scRNA-seq data to be used for genome-wide estimation of absolute transcript levels (*17*–*19*).

To determine whether high transcript accumulation rates are common across early *C. elegans* zygotic genes and to characterize rates genome-wide, we asked whether read counts in embryonic scRNA-seq data are predictive of absolute transcript levels in *C. elegans* embryonic cells. Comparing rates inferred from scRNA-seq with smFISH as a gold standard, we showed the utility of scRNA-seq in identifying high-rate genes. We found that genome-wide distributions of transcript accumulation rates vary substantially across cell types and lineages but are highest at the eight-cell stage of development. High-rate genes tend to have more recent duplicates and are enriched in chromosomal clusters, indicative of a rapidly evolving class of genes. We identified gene features associated with rapid accumulation, including short primary transcript length and lower intron size and count. Promoters of high-rate genes include binding sites for lineage-specific TFs and are enriched for the Initiator (Inr) motif, but individual core promoter motifs contribute only incrementally to rapid transcription of two exemplar high-rate genes, *ceh-51* and *sdz-31*. Our results suggest that rates are regulated at multiple levels, consistent with the robustness of *C. elegans* embryogenesis.

## RESULTS

### Analysis of scRNA-seq data reveals differences in transcript accumulation rates in the early *C. elegans* embryo

**Fig. 1.**
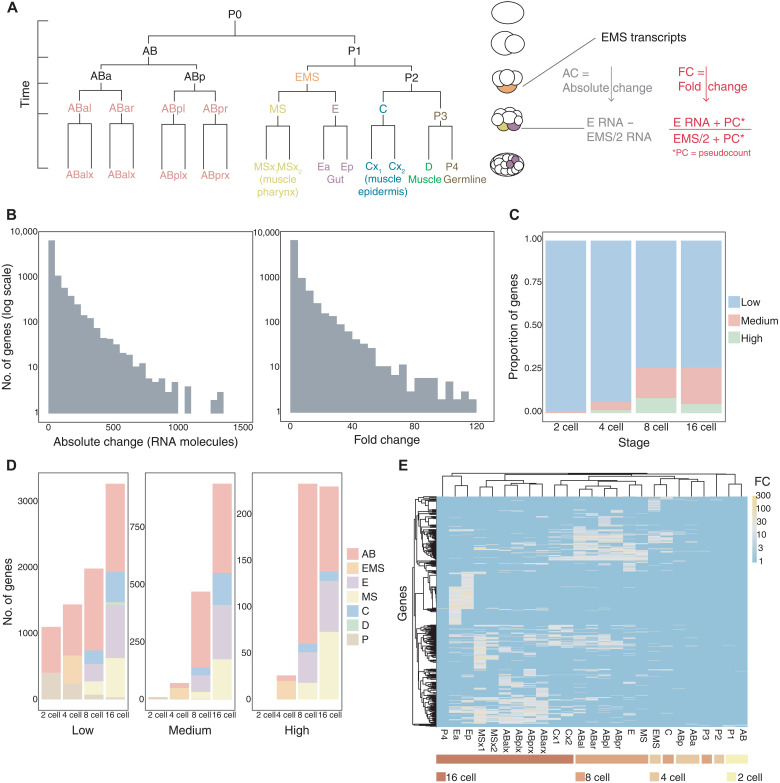
Estimating absolute transcription rates from scRNA-seq data. (**A**) Lineage tree of the *C. elegans* embryo up to the 16-cell stage showing all the founder cells (AB, EMS, MS, E, C, D, and P) and simplified list of the tissue types that they produce. Accumulation rates were determined by change in the number of transcripts in daughters compared to the parent cell (see Materials and Methods). (**B**) Histogram distribution of AC and FC for all genes and cells. (**C**) Fraction of the transcriptome at each embryonic stage based on rate category: high, medium, and low. (**D**) Number of genes in each rate category by embryo stage and main founder lineages. (**E**) Heatmap of all high-rate genes (high and medium) using average method of clustering with Pearson distances. Colored bars show embryo stage.

We predicted genes with high transcript accumulation rates by analyzing an existing scRNA-seq dataset that includes cells up to the 16-cell stage of the *C. elegans* embryo (Fig. 1A; see Materials and Methods) (*20*). In *C. elegans*, a first wave of zygotic genome activation occurs at 4-cell stage, leading to the establishment of the main founder lineages by the 16-cell stage. We estimated total RNA counts in each cell by correcting the raw pseudobulk transcripts per million (TPM) expression values for cell volume, on the basis of previous work showing that total mRNA content scales with cell size, including in *C. elegans* embryos (*20*–*22*). Comparing the TPM values and smFISH counts for the previously studied endodermal genes led us to use an estimate of 8,000,000 mRNA molecules per embryo (*12*, *20*), which we also used to calculate the final absolute RNA counts. We used two metrics to identify rapidly accumulating zygotic transcripts. First, we calculated the absolute change (AC) for each gene by subtracting the number of transcripts in each cell from those predicted to be contributed by its mother (Fig. 1A, Materials and Methods). This provides an estimate of the number of new transcripts produced during each cell cycle. Second, we used the fold change (FC) from parent to daughter to distinguish newly transcribed genes from those already present at high levels in the parent. Last, we removed maternally expressed genes [TPM, >12.5 in the one-cell embryo stage (P0)] to limit our analysis to zygotically transcribed genes.

By combining both AC and FC metrics, we identified newly transcribed genes with high rates of transcript accumulation. To identify common features associated with different rates of accumulation and study rate regulation, we broadly categorized genes on the basis of their AC and FC (Fig. 1B). We classified genes on the basis of a loose binning strategy as “high rate,” having at least 200 new transcripts and FC > 10; “medium rate,” having at least 50 new transcripts and FC > 5; and “low rate,” having <50 new transcripts and FC < 5 (Fig. 1B and fig. S1B). The majority of zygotically expressed genes showed low accumulation rates across all early embryonic stages tested, but a substantial fraction accumulates at high (8.7% at the 8-cell stage and 5.2% at the 16-cell stage) or medium rates (17.5% at the 8-cell stage and 21.2% at the 16-cell stage) (Fig. 1C and fig. S1D). We confirmed that at these thresholds, the number of genes predicted to have high or medium rates in germline (P) cells was very low, consistent with the known transcriptional quiescence of these cells (Fig. 1D and fig. S1A) (*23*). In total, we predicted 205 high-rate and 524 medium-rate genes. Zygotic genome activation in *C. elegans* begins at the 4-cell stage (*24*, *25*), and we detected the most high- and medium-rate genes at the 8- and 16-cell stages (Fig. 1, C and D). At these stages, high-rate transcription is detectable in most somatic cells except the D blastomere, which may have been collected for single-cell analysis too soon after its division from the germ line to have activated transcription (Fig. 1D). Overall, the AB and E lineages express the largest number of genes with high and medium rates, followed by the MS and C lineages (Fig. 1D and fig. S1, C and E). Hierarchical clustering based on AC of high- and medium-rate genes grouped cells by embryo stage and lineage, likely reflecting similarities in genes that need to accumulate rapidly in related cells (Fig. 1E).

### Validation of accumulation rates using smFISH

**Fig. 2.**
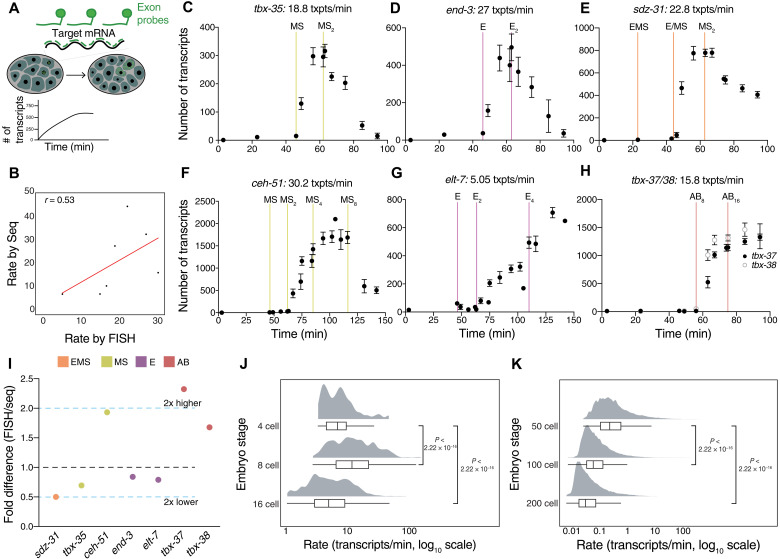
Comparison of inferred rates from scRNA-seq with smFISH. (**A**) smFISH method used for measuring accumulation rates. Nuclear staining was used to count number of cells to estimate time. (**B**) Correlation between estimated rates from scRNA-seq and smFISH (Pearson correlation). (**C** to **H**) smFISH counts (for seven different genes in different lineages) over developmental time used to calculate transcript accumulation rates between the two cell divisions of maximal expression. (**I**) Fold difference in estimated rates by smFISH and scRNA-seq for the seven genes tested. Dashed blue lines show ±2-fold difference. (**J**) Distribution of transcript accumulation rates from scRNA-seq estimates (AC/min) for all high- and medium-rate genes across cell types in the AB, EMS, E, MS, and C lineages at the 4-, 8-, and 16-cell stage. (**K**) Distribution of transcript accumulation rates from scRNA-seq estimates (AC/min) at later embryo stages estimated from the dataset of Packer *et al.* (*30*). In (J) and (K), adjusted *P* values are from Wilcoxon test.

Because scRNA-seq measurements can be noisy (*17*), we tested the validity of our rate predictions by an orthogonal method, smFISH. In this method, we targeted multiple oligonucleotide probes to the exons of target mRNAs, allowing single transcripts to be visualized as diffraction-limited spots (Fig. 2A and fig. S2A) (*14*). The spots can be counted to determine the absolute number of mature transcripts in each embryo. We determined the stage of each embryo by counting 4′,6-diamidino-2-phenylindole (DAPI)–labeled nuclei (Fig. 2A). We found that all (seven of seven) candidate high- and medium-rate genes that we tested showed corresponding high transcript accumulation by smFISH (most within twofold difference in rate; Fig. 2, C to I). The rates estimated by the two methods show a modest positive correlation (*r* = 0.53; Fig. 2B). One major contribution to the discrepency likely comes from staging differences, which is more fine-grained in our smFISH measurements. However, this level of correlation is similar to previous comparisons between different scRNA-seq platforms and smFISH counts in melanoma cell lines (*17*). We infer from this analysis that the majority of high-rate genes identified from scRNA-seq are being correctly classified by our approach.

### Maximum transcription rates vary across stages

*C. elegans* embryonic cells change markedly across stages. The embryo undergoes reductive cleavage, with cell and nuclear volumes decreasing by about twofold each division (*26*). Meanwhile, cell cycle length increases over time, with each cell dividing slower than its mother (*27*, *28*). Last, maternal repressors prevent transcription at the one-cell and two-cell stages, and the degradation of these repressors over time in somatic lineages facilitates the onset of zygotic transcription at different times in different lineages (*29*). This raises the question of whether maximum transcription rates vary by stage or lineage.

To ask whether there are genome-wide differences in transcript accumulation rate between stages and lineages, we calculated the accumulation rate of all high- and medium-rate genes detected in the scRNA-seq data. We found that while high-rate transcription occurs at the 4-cell, 8-cell, and 16-cell stages, concordant with our smFISH imaging, the overall distribution of rates is highest at the 8-cell stage (Fig. 2J). This seems broadly true across most lineages; the median accumulation rate of medium- and high-rate genes is higher for cells at the 8-cell stage compared to their daughters at the 16-cell stage (fig. S2B). Overall median rates at the 8-cell stage are about two- to threefold higher compared to the 4-cell and 16-cell stages (fig. S2C). To contrast this with later development, we estimated the absolute rate change in later cells from the scRNA-seq dataset generated by Packer *et al.* (*30*). The estimated rates at the 8-cell stage are 49-fold higher than the rates for cells born around the 50-cell stage, and rates continue to decline at the 100- and 200-cell stage (Fig. 2K). Within these later stages, rates are fairly consistent across lineages (fig. S2D). We conclude that the extent of high-rate transcription varies between embryo stages, with an apparent peak at the eight-cell stage, very quickly after the onset of zygotic transcription.

To further examine absolute transcription rate differences between lineages, we used our smFISH measurements of genes expressed in the MS (mesoderm), E (intestine), and ABa (ectoderm and pharynx) lineages (Fig. 1A). The MS cell expresses *tbx-35*, which encodes a T-box TF required for normal mesodermal development (*31*). Between the birth and the division of the MS cell (16 min), ~300 transcripts of *tbx-35* accumulate, giving an accumulation rate of ~19 transcripts/min (Fig. 2C). *ceh-51*, which is expressed one cell cycle later in the MS daughter cells and is activated by TBX-35 (*32*), accumulates at an even higher rate of ~30 transcripts/min (Fig. 2F). *end-3*, which encodes a GATA TF involved in intestinal fate specification, is expressed in the E cell but, at the same time as *tbx-35*, has a similarly high rate of 27 transcripts/min (Fig. 2D). However, *elt-7*, a main target of *end-3* in the E daughter cells (*33*), has a much lower accumulation rate (~5 transcripts/min; Fig. 2G). Although *elt-7* accumulates to a fairly high transcript level (~500 total transcripts) during the E2 cell cycle, the length of this cell cycle is much longer. The transcripts of *sdz-31*, a predicted transmembrane protein (*34*), accumulate in both E and MS cells at 23 transcripts/min per cell (Fig. 2E). Last, we also imaged *tbx-37* and *tbx-38*, two paralogous genes expressed early in the ABa lineage and play important fate specification roles in ABa descendants (*35*, *36*). *tbx-37/38* each accumulate at ~16 transcripts/min in ABa granddaughters (Fig. 2H). Our smFISH analysis indicates that RNA accumulation rates can vary from gene to gene and that the AB, E, and MS lineages are capable of high-rate transcription.

### Transcript synthesis is an important contributor to total accumulation rates

**Fig. 3.**
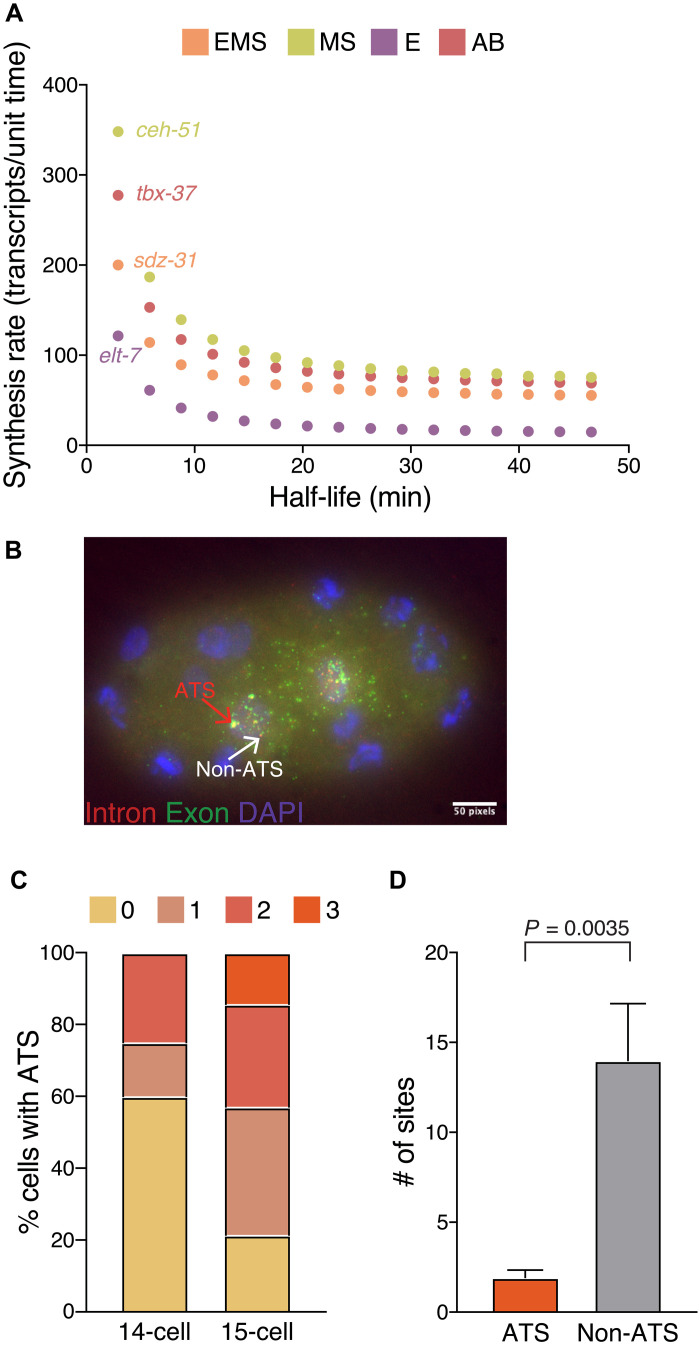
Decay and nascent transcription dynamics. (**A**) Estimated mRNA synthesis rates from a range of mRNA half-lives (*t*_1/2_). *t*_1/2_ range was set from decay estimates from whole-embryo transcript measurements [see fig. S3 (A to D)] (*37*). (**B**) Maximum intensity projection of 15-cell embryo with *ceh-51* introns labeled with cyanine5 (cy5) (red) and exons labeled with cyanine3 (cy3) (green), DAPI marks nuclei. Distribution of intensities for individual embryos was used to classify ATS: brightest intron-exon colocalized spots (red arrow) and lower-intensity colocalized spots are classified as non-ATS (white arrow). (**C**) Percent cells at the 14- and 15-cell stages that have 1, 2, 3, or 4 ATS. (**D**) Number of cells in the 14- and 15-cell embryo stages combined with ATS compared to non-ATS. *P* value was from Mann-Whitney test. *N* = 18 embryos for (C) and (D).

Absolute transcript levels are representative of both transcript production and degradation. As there are presently no direct genome-wide measurements of zygotic transcript half-lives (*t*_1/2_) in the *C. elegans* embryo, we can place limits on half-lives from whole-embryo time course RNA-seq data (*37*). We estimated *t*_1/2_ for genes with sufficient data points for a one-phase decay fit (example gene in fig. S3A) and found that genes with higher accumulation rates tend to have shorter half-lives (fig. S3B). Taking these half-lives into account gives adjusted synthesis rates slightly higher than the raw accumulation rates for medium- and high-rate genes, with a median of 1.3-fold higher across cells (fig. S3, C and D). For four candidate genes, we also determined what synthesis rates would be required for the observed increases in mRNA levels given a range of plausible half-lives (Fig. 3A). These synthesis rates varied only modestly except in the case of extraordinarily low *t*_1/2_, overall indicating that mRNA accumulation rates are likely a good proxy for transcript production rates for most genes (Fig. 3A).

Depending on the replication status, a cell can have two to four DNA loci, and all or none of the loci could be actively transcribing at a given time. For one high-rate gene, *ceh-51*, we determined how many loci were active in individual cells. We imaged nascent transcription, or active transcription sites (ATSs), observed as high-intensity colocalized intron-exon spots. When *ceh-51* mRNA first appears, in the MS daughter cells of 14-cell stage embryos, 40% of these cells have either one or two detectable transcribing loci, while the remainder have none (Fig. 3C). At the 15-cell stage, which begins 4 min later, 80% of MS cells have from one to three ATSs (Fig. 3C). In cells with more than one ATS, the intensities of individual exon or intron spots within an ATS were similar (fig. S3E). These results are consistent with individual genomic loci contributing equally and independently to nascent transcription and *ceh-51* RNA levels.

In addition to ATS, we identified a significant number of exon-intron colocalized nuclear spots with lower intensities than the nascent transcription sites (Fig. 3, B and D). These spots likely represent unspliced transcripts that have not been exported out of the nucleus, as have been identified in other systems (*38*).

### High-rate genes include dosage-sensitive TFs

**Fig. 4.**
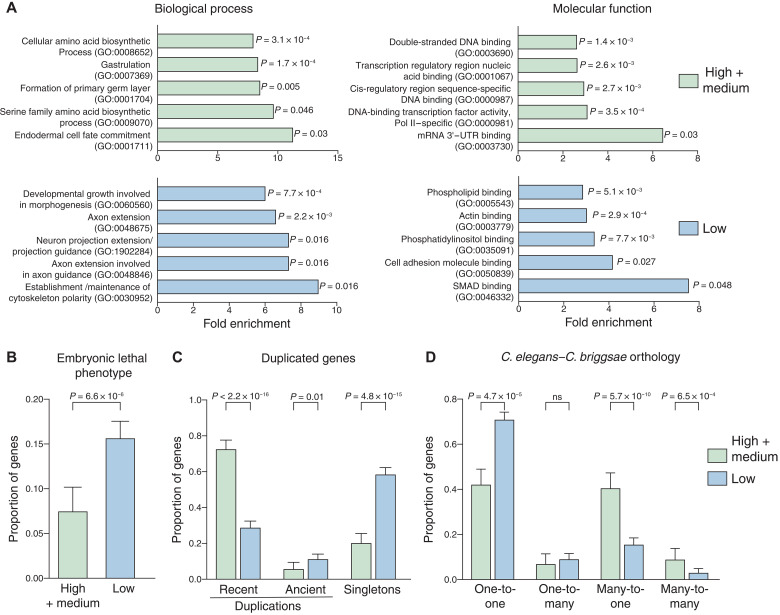
Function and evolutionary conservation of high-rate versus low-rate genes. (**A**) Gene ontology categories that show enrichment within high- and medium-rate (combined) and low-rate genes, from PANTHER analysis. Top five GO terms shown for each representative terms were used for similar categories. False discovery rate (FDR)–adjusted *P* values are shown. (**B**) Proportion of all high- and low-rate genes with embryonic lethal phenotypes. (**C**) Proportion of all high- and low-rate genes that have duplications or are singletons [using data from the work of Ma *et al.* (*56*)]. (**D**) Orthogroups identified between *C. elegans* and *C. briggsae* in all high-rate versus low-rate genes. Error bars, 95% confidence intervals. All reported *P* values from Fisher’s exact test.

To understand what types of genes are rapidly transcribed in the early embryo, we performed Gene Ontology (GO) analysis on high- and medium-rate (high + medium) and low-rate genes to identify biological processes and molecular functions associated with each rate group. High- and medium-rate genes are enriched for annotations associated with the molecular function categories of TFs (DNA binding activity) and mRNA binding [3′ untranslated region (3′UTR) binding] and bioprocess terms related to development (cell fate specification and gastrulation; Fig. 4A). By contrast, low-rate genes are enriched for terminal cell type–specific annotations and housekeeping functions (Fig. 4A). Ten percent of high- and medium-rate genes, including known fate specification regulators, are annotated as TFs in the wTF3.0 worm TF database versus 3% of low-rate genes (table S1) (*39*). We also find high-rate TFs with lineage-specific expression (e.g., *ceh-76* in the early MS lineage, *sdz-12* in the ABal lineage, and *ets-7* that shows posterior-specific expression in multiple lineages (*40*, *41*), but the specific roles of these factors in fate specification are so far unstudied, making them exciting candidates to be validated in the future.

We asked whether rapidly transcribed genes were more likely to be essential by testing for enrichment of embryonic lethal mutant or RNA interference (RNAi) phenotypes as curated by WormBase (*42*). Unexpectedly, the proportion of low-rate genes with embryonic lethal phenotypes was significantly greater than the high- and medium-rate genes (Fig. 4B). The essentiality of low-rate genes could reflect their enrichment for housekeeping functions, and hence, they might more likely be evolutionarily conserved over longer distances. Using the OrthoList2 database of worm-human orthologs (*43*), we found that a significantly greater proportion of low-rate genes had a one-to-one human ortholog compared to medium- and high-rate genes (fig. S4A).

Because several of the high-rate genes (e.g., *end-3*, *elt-7*, and *tbx-37*) are known to have partially redundant paralogs, we asked whether high-rate genes were enriched for such gene duplications. Consistent with this, high-rate genes are significantly more likely to have been recently duplicated (threefold enriched compared with low-rate; Fig. 4C) and have a predicted paralog (fig. S4B), while more low-rate genes occur as singletons (Fig. 4C). Because high-rate genes were more likely to have recent duplicates in *C. elegans*, we asked whether they were also more likely to have multiple orthologs in the related species *Caenorhabditis briggsae *(*44*). Although they diverged ~30 million years ago, both species are morphologically and behaviorally similar (*45*). We found that low-rate genes were 1.7-fold more likely to have one-to-one orthologs in *C. briggsae* compared to high- and medium-rate genes (Fig. 4D). However, greater proportion of high- and medium-rate genes have many-to-one (2.5-fold over low rate) or many-to-many orthologs (2.7-fold over low rate) in *C. briggsae* (Fig. 4D).

We conclude that high-rate genes are important developmental regulators and that rapid transcription is associated with increased rates of gene copy number evolution. Seventy-five percent of high-rate genes have paralogs that are also high rate compared with 1.8% of all genes that have paralogs that are high rate (fig. S4C), further suggesting that redundancy between paralogous high-rate genes may mask their phenotypes.

### High-rate genes share gene-specific structural features and form genomic clusters

**Fig. 5.**
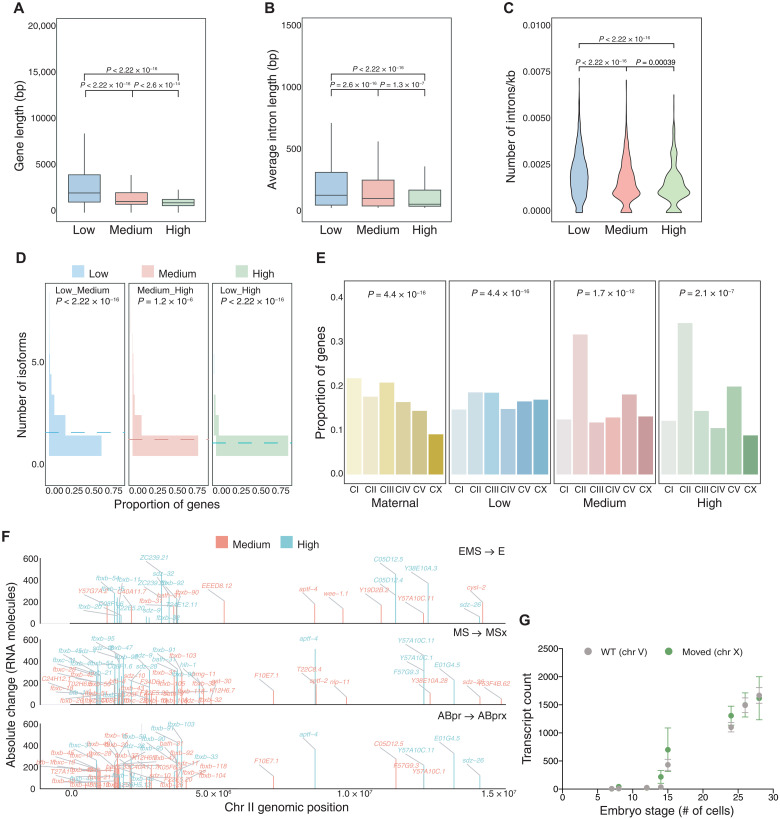
Genetic architecture and features associated with high transcript accumulation. (**A** and **B**) Gene and intron lengths across different rate categories. (**C**) Length-normalized intron number in each rate category. (**D**) Distribution of isoform number based on proportion of genes in each rate category. The dotted line shows the mean. All reported significance was from Wilcoxon test with adjusted *P* values. (**E**) Number of genes (only protein coding included) as a proportion of the genes in each category found on the different chromosomes. FDR-adjusted *P* values from chi-square test for a comparison of how different the distribution is for each category compared to all genes. (**F**) Karyogram of genes with FC > 5 on chromosome II showing AC for each gene (medium or high rate) across genomic position in the indicated cells compared to their parent. (**G**) smFISH counts for *ceh-51* over developmental time comparing *ceh-51* at its native location [wild type (WT)] with the gene moved to the X chromosome.

Previous studies in other organisms have shown that early zygotic genes tend to be shorter and have shorter introns compared to maternally expressed genes (*46*). We tested whether this was true for highly transcribed zygotic genes in *C. elegans* embryos and found that high- and medium-rate genes have shorter primary transcript length than low-rate genes (Fig. 5A). The presence of introns has been linked to higher transcription efficiencies (*47*, *48*), but we found that higher rate is also associated with shorter introns (Fig. 5B). We calculated the number of introns for each rate category (adjusted for gene length) and found a decreasing trend between rate and intron number (Fig. 5C). We also found that high- and medium-rate genes tend to have fewer annotated isoforms (Fig. 5D) and are significantly less likely to be trans-spliced (about threefold difference between high- and low-rate genes; fig. S5A) (*49*, *50*). Our findings suggest that such short, intron-poor, genes are enriched in the earliest zygotic stages and are consistent with the intron delay hypothesis, which posits that longer introns preclude high levels of expression in early development when cell cycles are short (*51*, *52*). These results are also consistent with known classic examples of intronless genes in worms, the MED TFs, which encode the shortest known GATA factors and are critical for endomesodermal (EMS) fate specification (*53*).

Position effects, where chromosomal position influences gene expression, are widespread in many species (*54*). To ask how chromosomal position correlates with transcript accumulation rates, we analyzed the distribution of genes in each rate category across chromosomes and found an overrepresentation of high- and medium-rate genes on chromosome II (Fig. 5E and fig. S5B). Examining the position of high-rate genes within each chromosome shows large clusters of rapidly transcribed genes on the very left arm of chromosome II (Fig. 5F, example cells E, MSx, and ABprx). Most of these clustered genes are either in the F-box family or the *bath* (BTB and MATH domain containing) family, both of which are substrate-binding adapters for ubiquitin-mediated proteolysis and are rapidly evolving in *Caenorhabditis* (*55*). More clusters of genes are seen on chromosome II compared to other chromosomes in several cell types, perhaps reflecting the large cluster of F-box genes that is enriched on this chromosome (fig. S6, A to C) (*56*). When the F-box genes and those in the BTB, MATH, and SKR family involved in ubiquitin-mediated proteolysis are removed, the remaining high-rate genes are still enriched but not significantly on chromosome II, although a significant enrichment emerges on chromosome V (fig. S5, C and D). Last, we see that accumulation rates are higher for genes on autosomes compared with the X chromosome, consistent with reduced expression from the sex chromosome in the germ line and early embryo (fig. S5E).

To test whether genomic context influences rate regulation, we used a Mos transposon approach (*57*) to move the entire high-rate gene *ceh-51* along with 800 bp of the cis-regulatory (region upstream of the translation start site) to a random site on the X chromosome (~8.4 Mb). The accumulation rate and RNA levels of *ceh-51* at this new location were very similar to *ceh-51* in the native context (Fig. 5G), suggesting that the larger region surrounding the *ceh-51* gene is not essential for its high rate.

These results indicate that high-rate transcript accumulation is correlated with specific gene architectures and genomic location. Whether chromosomal clustering, the absence of trans-splicing, shorter genes, and fewer isoforms mechanistically facilitate high-rate transcription or are correlated for other reasons should be explored further in the future.

### The Inr motif is enriched in promoters of high-rate genes

**Fig. 6.**
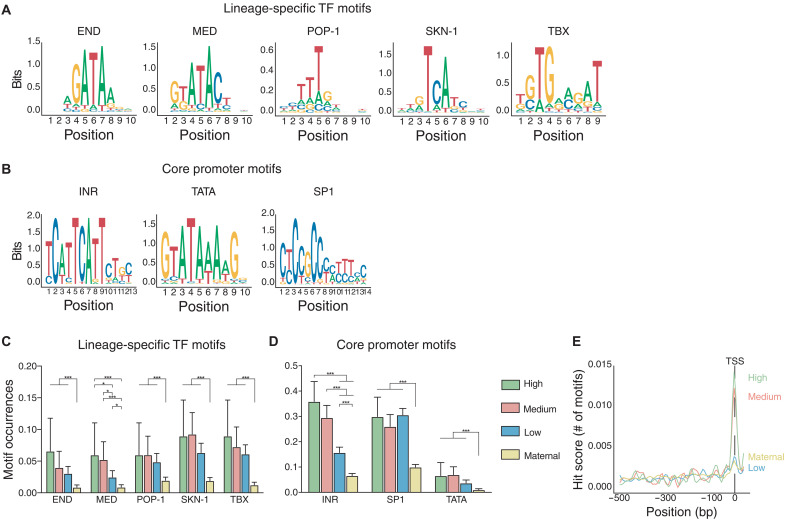
Promoter motifs associated with rapid transcription. (**A** and **B**) Position scores of lineage-specific (A) and core promoter (B) motifs. Inr and SP1 motifs from the MEME suite, TATA motif from Chen *et al.* (*59*), and lineage-specific TF motifs from CIS-BP (http://cisbp.ccbr.utoronto.ca/). (**C** and **D**) Motif occurrences for the lineage-specific TFs in A (C) or core promoter elements in B (D) across different rate categories and maternal genes (95% confidence intervals as error bars, *P* values from chi-square test, and FDR-adjusted *P* values) *P ≤ 0.05, ***P ≤ 0.001. (**E**) Inr motif distribution across the promoter (500 bp upstream) in genes from the indicated rate categories showing overlap of Inr with the TSS.

We next asked what cis-regulatory elements play a role in controlling accumulation rates. *C. elegans* has a small genome with most cis-regulatory elements within a few kilobase of the transcription start site (TSS) (*58*). A few early zygotic promoters have been studied and found to be largely controlled by the promoter-proximal regions rather than distal enhancers (*53*). To find promoter motifs that might control rapid transcription across different cell types, we first combined three different datasets (*49*, *59*, *60*) to identify the most likely TSS (see Materials and Methods). We then asked what sequence motifs were enriched in high- and medium-rate gene promoters (operationally defined as the 500 bp upstream of the TSS). We tested both motifs identified by the de novo motif-finding program MEME, which included the well-studied core promoter motifs Inr and binding sites for the SP1 TF (SP1), also the TATA box (Fig. 6B). We also examined the binding sites for known early lineage-specific TFs (END, MED, POP-1, SKN-1, and TBX; Fig. 6A). We found that these regulators were enriched in high-rate promoters active in the expected cells. For example, the SKN-1 binding site was enriched in high-rate promoters expressed in the EMS cell (fig. S7A), and the MED and END motifs were more likely to be found in promoters active in the E cell or its daughters (fig. S7, B and C). In addition, as expected, binding sites for these regulators are mostly found in early embryonic genes across all three rate categories in contrast with maternal genes (Fig. 6C). However, there is a significant collective enrichment of sites for these five lineage-specific TFs (Fig. 6A) in both high- and medium-rate genes compared to low-rate genes, indicating that they may be important for rate regulation (fig. S7D).

Among the core promoter motifs, the initiator element (Inr) stood out as strongly associated with rate (Fig. 6D). Transcription of many *C. elegans* genes has been previously shown to initiate at the “A” of the tcAttc core Inr motif (*59*). Inr motifs had significantly greater occurrence in high-rate genes compared with medium-rate genes and in both categories compared to low-rate and maternal genes (Fig. 6D). Furthermore, the Inr motif was strongly enriched at the TSS of high- and medium-rate genes, but this enrichment was lower for low-rate or maternal genes (Fig. 6E). Other core promoter motifs including the TATA box and binding sites for the general TF SP1 are highly but uniformly enriched in low-, medium-, and high-rate genes relative to maternal genes (Fig. 6D), suggesting that distinct core promoter structures may distinguish zygotic from maternal gene expression.

### Multiple promoter motifs contribute to accumulation rates

**Fig. 7.**
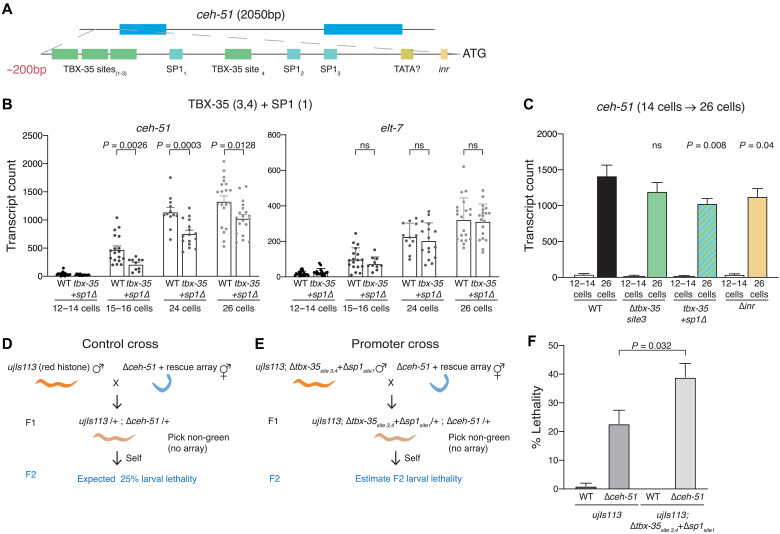
Contribution of promoter motifs to regulation of *ceh-51* transcript accumulation rate. (**A**) Two hundred–base pair upstream region (from translation start ATG) of *ceh-51* showing important motifs. (**B**) smFISH exon counts of *ceh-51* and *elt-7* at the indicated stages in the promoter mutant where TBX-35 sites 3 and 4 and SP1 site 1 are deleted (*N* = at least 3 biological replicates; *P* values from Mann-Whitney test). (**C**) Accumulation rate of *ceh-51* (from the 14-cell stage to the 26-cell stage) in the indicated mutants (*N* ≥ 3 biological replicates; *P* values from glm model of change in rates). (**D** and **E**) Genetic cross strategy to test the effect of reduced rate in the *ceh-51* (*tbx-35* sites 3 and 4 *+ SP1* site 1) promoter mutant. Red histone males [control in (D) or promoter mutant in (E)] were crossed to *ceh-51* homozygous deletion hermaphrodites. F1s without the *ceh-51* rescuing array were singled, and 20 to 25 embryos were used to score L1 survival of F2 animals. (**F**) F2 larval lethality from cross shown in (D) and (E). Cross to WT animals carrying the *ceh-51* rescuing array used as an additional control. Data are means ± SEM from *N* > 6 biological replicates. *P* values are from unpaired *t* test.

We tested the importance of TSS-proximal motifs for transcript accumulation rates of two high-rate genes: *ceh-51* and *sdz-31*. The T-box TF TBX-35 is known to regulate *ceh-51*, and four binding sites for TBX-35 required for expression were previously identified in the upstream region (promoter) of *ceh-51* (Fig. 7A) (*32*). We used CRISPR to mutate TBX-35 binding sites in the endogenous *ceh-51* promoter. We obtained a 14-bp deletion that disrupts the core of the third TBX-35 site (fig. S8A) and used smFISH to determine whether the accumulation rate of *ceh-51* requires this site. Loss of this TBX-35 site resulted in a rate accumulation rate change of 4.4 compared with wild type (WT) from the 14-cell to the 26-cell stage, which is the stage when *ceh-51* has its maximal accumulation rate (Fig. 7C). A larger deletion that disrupts two TBX-35 sites (sites 3 and 4) along with an intervening SP1 site (fig. S8A) resulted in a significant reduction in *ceh-51* rate (26%; Fig. 7, B and C). As an internal control, we measured *elt-7* mRNA, which is expressed at the same time as *ceh-51* but in different cells, and found *elt-7* levels to be similar to WT in this promoter mutant (Fig. 7B). The *C. elegans* homolog of SP1, SPTF-3, is a general TF that has been previously shown to be required for proper expression of gut specification genes and the regulation of gastrulation (*61*). Our observation that SP1 sites are enriched in all zygotic rate classes relative to maternal genes (Fig. 6D) suggests that SP1 may be especially important during embryonic development. Consistent with this, knockdown of maternal *sptf-3* by RNAi leads to partially penetrant embryonic and larval arrest (fig. S8B). However, *sptf-3* RNAi did not significantly change the viability of the single TBX-35 site mutant (fig. S8B).

On the basis of the enrichment and close overlap of the Inr motif with the TSS of high-rate genes, we hypothesized that the Inr may be required for both expression pattern and rate of high-rate genes. To test this, we deleted the core motif of the Inr element in the *ceh-51* promoter and unexpectedly found only a modestly significant 21% reduction in the *ceh-51* accumulation rate (Fig. 7C). Deletion of the Inr element in a second high-rate gene, *sdz-31*, expressed in the early EMS lineage (Fig. 2E) had no effect on transcript accumulation (fig. S8, D to F). Thus, the Inr motif is dispensable for high-rate transcription for at least two genes. We suggest that high rates of transcript accumulation in the early embryo are likely under partially redundant control, such that not a single motif but a combination of cis-regulatory elements together specify the rapid accumulation.

To test the functional importance of *ceh-51* rates, we measured viability as a function of transcript dose. Homozygous *ceh-51* null mutants arrest as L1 larvae (*32*). In *ceh-51* promoter mutants with 15% (single TBX-35 site disrupted) or 21% (Inr deletion) reduction of transcript accumulation rate, L1 larval survival is near 100% (fig. S8C). To test whether lower levels of transcripts might affect phenotype, we measured the viability of trans-heterozygous mutants (Fig. 7, D and E). Self-progeny from *ceh-51* deletion heterozygotes are predicted to be ¼ viable WT, ½ null heterozygotes with a 50% reduction in *ceh-51* expression, and ¼ inviable null homozygotes (Fig. 7D). We found that this group had 22.7% L1 arrest (expected ¼), suggesting close to 100% viability if 50% of *ceh-51* expression is lost in mothers (Fig. 7F). Next, we examined self-progeny of mothers carrying one null allele and one promoter mutant disrupting two TBX-35 sites and one SP1 site (Fig. 7E). These are predicted to be ¼ viable promoter mutant homozygotes, ½ trans-heterozygotes with ~63% reduction in *ceh-51* expression, and ¼ inviable null homozygotes. We found that a significantly higher fraction of these progeny arrest as L1s (39%; *P* = 0.032), suggesting that a ~63% reduction of *ceh-51* levels results in increased lethality (Fig. 7F). We conclude through this genetic analysis that reduced-rate promoter mutant fails to fully complement a null allele of *ceh-51*, indicating the importance of rates in rates in MS lineage specification.

## DISCUSSION

### scRNA-seq as a tool to predict absolute transcript levels

Our comparisons with smFISH counts indicate that the estimation of absolute transcript levels can be inferred from scRNA-seq data with reasonable accuracy. Similar correlations, with disagreements typically in the ±2-fold range, were seen in previous comparisons of smFISH and scRNA-seq in human cell lines, suggesting that they may be generalizable across species and cell types (*17*). The actual agreement may be higher as we can only infer the correspondence between the stages analyzed by scRNA-seq and smFISH within a few minutes using nuclear counts. Thus, the correlation between imaging and sequencing counts during development could potentially be improved by more precise staging and collection of cells for sequencing. Recently developed high-throughput FISH methods such as seqFISH+ or MERFISH, which can image up to 10,000 genes, report strong correlations between single-molecule transcript counts and RNA-seq (*18*, *62*). However, imaging methods still require the design of expensive probe sets. Thus, comparing smFISH counts and scRNA-seq reads or unique molecular identifiers (UMIs) in multiple model systems should help develop principles for inferring absolute transcript levels from sequencing data.

### Importance of high transcript accumulation rates during embryonic development

Appropriate dosage of regulators is important for cell fate decisions during *C. elegans* embryonic development (*11*, *13*). TF dosage is similarly crucial for embryonic patterning in *Drosophila* (*15*) and in mammalian development and is slowly being recognized as underlying causes of human disease (*63*). We predict that transcript accumulation rates are key to achieving the final dosage of TFs within a critical time window, allowing for robust fate specification. We found that trans-heterozygous *ceh-51* mutants (one copy with reduced rate with a null second copy) showed phenotypes associated with cell fate specification defects of a critical muscle fate specification TF, supporting our hypothesis (Fig. 7F). We propose that our high-rate gene list provides several candidate TFs, whose exact role in embryonic fate decisions are yet to be determined.

We find that many high-rate genes are more likely to have been recently duplicated and have paralogs that could have partially redundant functions (Fig. 4C and fig. S4, B and C). These results support the hypothesis that rapid transcription occurs, in part, to increase the transcript dose for key developmental TFs. One explanation is that the required functional dosage of transcripts is not easily achievable from a single gene and combined transcripts from paralogous genes are required to reach the appropriate threshold level. The significant enrichment of high-rate genes in the many-to-many orthology group indicates that the need for duplicate genes to achieve rapid transcription might be conserved in *C. briggsae*, which could lead to independent gene duplications (Fig. 4D). We also find that clusters of high-rate genes (for example, on chromosome II) belong to the same gene family, suggesting that they may have arisen by local duplication of ancestral high-rate genes (Fig. 5, E and F) (*56*). Genes known to regulate gut specification, such as the *med-* and *end-*GATA factors, tend to exist as multiple paralogs across evolution in species closely related to *C. elegans*, and these genes also frequently exhibit synteny (i.e., are located in proximity on the same chromosome) across species (*53*). Whether the clustering of high-rate genes is solely due to this tendency of duplicated genes to form local clusters or whether this clustering affects rate, such as by regulating recruitment of high-rate genes into spatial compartments such as transcription hubs, remains to be seen. Our observation that individual motifs make modest contributions to rate regulation for *ceh-51* and *sdz-31* could be explained by the existence of transcription hubs where the high concentration of general transcription machinery maximizes transcription output (*64*, *65*).

In addition to the developing embryo, there are many more contexts where cells have to respond quickly to changing environmental signals. The immediate early response genes are a classic example of rapid activation of factors triggered by a vast number of external challenges. Immediate early response factors have shorter primary transcripts and exons relative to genome-wide distributions (*66*). Mechanistic studies of how transcription rates are controlled during *C. elegans* embryogenesis could thus be broadly applicable to other systems and organisms where rapid transcription is required and may reveal common underlying principles of gene regulation.

### The influence of transcript decay and posttranscriptional processing on RNA output

We used whole-embryo RNA-seq data to estimate the contributions of synthesis and decay to accumulation rates, and our analysis suggests that transcript production has a larger impact on total rates (Fig. 3A and fig. S3, C and D). This is consistent with studies in mammalian cell culture systems, during the response to stimuli such as lipopolysaccharides or hypoxia, where transcript synthesis contributes more to changing RNA levels than degradation (*67*, *68*). Many of the regulators that we analyze here are transiently expressed, with most transcripts disappearing within one or two cell cycles of the time of maximal expression. In accordance with this, genes with higher total accumulation rates tended to have shorter half-lives (fig. S3B). However, there is a possibility for decay rates to be different during the appearance and disappearance phases. Although we estimate that de novo transcription rates are modestly higher than the observed total accumulation rates when adjusting for decay (fig. S3, C and D), more direct measurements of half-lives during the time of transcript increase versus decrease will determine more precisely how much higher and even more impressive the total accumulation rate can be.

Posttranscriptional processing including RNA splicing is another phase of gene expression where rates may be tightly controlled. We found a number of unspliced transcripts of the MS-specific gene *ceh-51* outside nascent transcription sites in early muscle cells, which could mean that posttranscriptional splicing plays a role in how quickly mature transcripts of *ceh-51* are produced (Fig. 3D). Direct RNA-seq has broadly revealed that many transcripts in diverse cells are not spliced cotranscriptionally (*69*), although the importance of posttranscriptional splicing and the rates of other processing steps during cell fate specification still needs to be worked out.

### Cell size and transcription rates

An important normalization for scRNA-seq counts to estimate absolute mRNA numbers is to account for cell volume. Transcription rates are also known to scale with cell size across organisms (*22*, *70*). The *C. elegans* embryo develops by reductive cleavage in a constant-volume eggshell with cells reducing about twofold in size with each developmental cell division. We see the highest maximal transcript accumulation rates in the 8-cell stage, where cells are larger compared to the 16-cell stage, suggesting that transcription rates in the *C. elegans* embryo may also be driven by cell size in this context (Fig. 2, J and K). Future work should focus on identifying mechanisms involved in this scaling and how the balance between reduction in cell size and the ramping up of zygotic transcription is achieved as early development progresses.

### Boundaries on the maximum achievable transcription rates

What are the theoretical limits on transcription rates? Several factors contribute to this, including the available number of PolII and other TF molecules, the rates of initiation and elongation, gene length, and pause sequences. For short genes such as *end-3* (~1.3-kb primary transcript), at the commonly assumed elongation rate of 1.5 kb/min (*71*), the entire gene length would need to be completely occupied by PolII complexes during maximum expression. smFISH analysis of early zygotic genes in the *Drosophila* embryo have also measured high rates of transcription, potentially nearing the steric limits of PolII, suggesting that rates approaching theoretical maximum could be a common feature of gene expression in rapidly dividing embryos (*3*, *4*, *7*). Elongation rates have not been directly measured in the *C. elegans* embryo, although elongation speeds as high as 6 kb/min are seen in mouse embryonic stem cells (*72*). It will be interesting to determine whether initiation and elongation rates are coordinately high for genes with high accumulation rates.

We have characterized transcription accumulation rates in a developing multicellular organism by devising an approach to analyze scRNA-seq data and find that the regulation of rates is highly complex. We hypothesize that high rates are required to achieve precision in final transcript levels, which drives fate specification. How this precision will translate to protein levels is still unclear. There is good concordance between spatiotemporal expression patterns of proteins identified by lineage analysis by four-dimensional imaging during *C. elegans* embryonic development and transcripts determined by scRNA-seq (*40*). However, inferring accurate protein levels from imaging data still remains a challenge. Whether it is the RNA or protein production rates that are limiting during cell fate specification can be addressed once quantitative single-molecule protein measurements become more accessible. Our study predicts that both RNA and protein levels ultimately play roles in regulating cell fate specification and overall developmental robustness.

## MATERIALS AND METHODS

### Accumulation rates from scRNA-seq data

All analysis was performed using the R statistical programming language. Analysis code can be found at github.com/p-sivaramakrishnan/C-elegans-rate-analysis. Gene names from the dataset of Tintori *et al.* were transferred to WS260 reference. We note that because the timing of division varies between lineages, the 16-cell stage in the Tintori dataset reflects the 16 cells of the AB lineage or the 24-cell stage of the embryo. For the sake of simplicity, this stage is still referred to as the 16-cell stage.

Median TPM counts for each gene in each cell were calculated; cells annotated as “tossed” were not used. Absolute change for each gene in each cell was calculated as (TPM_daughter_ − TPM_parent_) × volume of the daughter cell × 8,000,000. The 8,000,000 mRNA molecules per embryo is based on previous smFISH absolute counts measured for endodermal genes (*12*) and unchanging total mass of RNA for each early stage and determined from spike-in controls by Tintori *et al.* (*20*). Thus, the AC metric reflects the difference in transcript copy number between parent and daughter, adjusting for preexisting transcripts inherited by each daughter from the parent. FC between daughter and parent was calculated with a pseudocount of 10 (to get estimates even when TPM in the parent was 0). The FC metric was introduced to reduce measurement noise to exclude genes that are already at high levels in the parent cell and not changing by much (through new transcription) in the daughter cell. These could include highly expressed housekeeping genes that are ubiquitous in all cells. Genes with absolute transcript amounts >100 (TPM, 12.5) in the P0 cell were considered to be maternal genes.

### Rate categorization

Distributions of AC and FC (Fig. 1B) were used to bin rate categories. AC > 200 and FC > 10 were considered high rate; AC between 50 and 200 and FC between 5 and 10 were considered medium rate, and AC between 1 and 50 and FC between 1 and 5 were considered low rate. If the AC or FC was high but the other fell into the medium category, then it was classified as medium rate. Similarly, if AC and FC were medium or high but FC was low (1 to 5), then those genes were classified as low rate. For gene structure and motif analysis, only high- and medium-rate genes that were never low rate in any cell were included in the analysis.

### Half-life estimation

From whole-embryo time course RNA-seq data (*37*), using the time interval when TPM levels decrease from maximum expression to 0, we calculated half-lives from a one-phase decay fit. Half-lives were estimated for 4678 genes for which there were sufficient data points for a one-phase decay fit (see data file S1). Adjusted synthesis rates (*k*) were then calculated from these half-lives using the formula

$$A_{T}=(k/\lambda -A_{0})(1-e^{-\lambda t})$$

where *A_T_* and *A*_0_ are transcript abundance at time 0 and time *t*, *k* is the synthesis rate, and λ is the decay rate (=0.693/*t*_1/2_).

### GO and conservation with *C. briggsae*

GO was performed using PANTHER tools (http://geneontology.org/) using the Overrepresentation Test (Released 02 February 2022) against the *C. elegans* reference list and Fisher’s exact test with false discovery rate correction. OrthoFinder was used to examine conservation between *C. elegans* and *C. briggsae* (*44*). The longest transcript isoforms were used to identify Orthogroups, which were then categorized as having one-to-one, one-to-many, many-to-one, or many-to-many orthologous genes between *C. elegans* and *C. briggsae*.

### Paralogous genes and embryonic lethal phenotypes

Data on gene duplications were obtained from the work of Ma *et al.* (*56*). A list of paralogs (Blast results from an *e*-value threshold of 10^−15^) that were also syn-expressed was obtained from the work of Tintori *et al.* (*20*). WormBase SimpleMine was used to identify allele and RNAi mutant phenotypes (https://wormbase.org//tools/mine/simplemine.cgi).

### TSS refinement

We collated TSS data from three different datasets [Saito *et al*., Chen *et al.*, and Kruesi *et al.* (*49*, *59*, *60*)], where TSS was identified by different methods. This allowed us to predict the most likely single TSS for each gene. Promoter regions were then defined as 500 bp upstream of this newly consolidated TSS.

### Motif tools

Xstreme (Meme suite; https://meme-suite.org/meme/) analysis using a background Markov order of 2 and Homer tools (background adjusted to *C. elegans* promoters) were used for de novo motif prediction. The Find Individual Motif Occurrences (FIMO) tools in the Meme suite was used for motif enrichment using a *P* value cutoff of 1 × 10^−4^, and the R package ggseqlogo was used to plot motifs.

### Worm stains and growth

Worms were grown on standard Nematode Growth Medium (NGM) plates on OP50 bacteria. Isopropyl-β-d-thiogalactopyranoside (1 mM) and carbenicillin (50 μg/ml) were added for RNAi plates. For smFISH, worms were grown on large, enriched peptone plates seeded with NA22 bacteria. All growth was at 20°C unless otherwise stated.

### Single-molecule RNA FISH

smFISH was performed as described by Nair *et al.* (*12*). Briefly, adult worms were treated with alkaline bleach to release embryos, which were washed with M9 buffer. Embryos were fixed with 4% formaldehyde [in phosphate-buffered saline (PBS)] and permeabilized by immersing the tube in dry ice and ethanol bath for 1 min. After thawing and a 20-min incubation on ice, embryos were washed with PBS and stored in 70% ethanol at 4°C. For hybridization with FISH probes, first, the ethanol was washed off the embryos with wash buffer (10% formamide in 2× SSC with 0.1% Triton X-100). FISH probes were used at 2-4 nM concentration in 100 μl of hybridization buffer (0.1% dextran sulfate and 0.1% formamide in 2× SSC buffer). Hybridization was performed overnight at 37°C. Before imaging, embryos were stained with Hoechst stain for 30 min and mounted on a coverslip in 2× SSC. smFISH images were taken with a standard epifluorescence scope. For dual labeling, introns probes were labeled with cyanine5 (cy5) and exons with cy3.

### smFISH transcript counts

TIFF stack images were processed using MATLAB as in (*12*). Embryos were segmented, and a Laplacian of Gaussian filter was applied to isolate single transcripts. Transcript counts were thresholded manually to obtain spot counts across all relevant *Z* stacks. Each channel for different probe sets was separately thresholded. Hoechst stain was used to determine the number of nuclei and cell stage.

### CRISPR deletions

Promoter deletions were performed by CRISPR using short single-stranded guide RNAs [as described by Dokshin *et al.* (*73*)]. Guides (see data file S1 for sequence) and tracrRNA were obtained from Integrated DNA Technologies. *Streptococcus pyogenes* Cas9 was purchased from the QB3 MacroLab, University of California, Berkeley. Coinjection marker *punc*-119::GFP (green fluorescent protein) was used to screen for positive injections. GFP-positive F1s were singled, and deletions were confirmed by sequencing. For the *sdz-31* Inr mutant, the germline Cas9 strain was used (EG9882), and guide and tracrRNA were injected without Cas9.

### Integration of *ceh-51* at a random genome location using the miniMos system

The *ceh-51* gene (including intron) along with 800 bp upstream and 260-bp 3′UTR was cloned into the pCFJ1202 miniMos plasmid (*57*). The plasmid with the construct was injected at a concentration of 10 ng/μl along with the coinjection plasmids as per the miniMos protocol (*57*). Injected plates with one to three worms were placed at 25°C. NeoR selection was performed by adding 500 μl of G418 (25 mg/ml) directly to the plates on the next day. Worms were allowed to starve out by continuing growth at 25°C and then heat shocked at 34°C for 3 hours. The next day, live animals that have none of the fluorescent injection markers (loss of extrachromosomal array) were singled. Polymerase chain reaction (PCR) was performed to test for insertion. The location of the insert was determined using the inverse PCR protocol described by Frøkjær-Jensen *et al.* (*57*). Briefly, genomic DNA was isolated, digested with Dpn II, and then ligated. The first round of PCR was performed with primers oCF1587 and oCF1588, and the second PCR was performed with primers oCF1589 and oCF1590 after diluting the first PCR product 100-fold. Reaction from the second PCR was cleaned up and sent for sequencing with primer oCF1590. The location of the insert was identified using BLAST.

### Genetic cross strategy for estimating larval lethality in progeny of heterozygotes

Promoter mutant (Δ*tbx-35_site3,4_ + SP1_site1_*) strain containing the red histone marker (from JIM113 - [pie-1p::mCherry::H2B::pie-1 3′UTR + nhr-2p::his-24::mCherry::let-858 3′UTR + unc-119(+)]) was generated by crossing JIM113 males with the promoter mutant hermaphrodite. As described in Fig. 7 (D and E), this strain (or JIM113 alone) was then either crossed to the Δ*ceh-51* null deletion mutant (MS1206) carrying an extrachromosomal array [*ceh-51*(+) + *unc-119::CFP* + *rol-6(su1006)*] or WT carrying the same array as a control. Twenty to 25 F1 embryos that had red fluorescence (indicating cross progeny) were transferred to a new plate and allowed to grow until the L4 stage. Single hermaphrodite L4s that were cyan fluorescent protein–negative (lacking the rescuing array) were transferred to a new plate. Twenty to 25 F2 embryos were then transferred to a new plate the next day. The viability of the F2 was scored as embryonic lethal, larval lethal, or L4 survival. L1 larval lethality was calculated as the number of dead L1s as a proportion of hatched worms.
