# Supplementary material for: Transcript accumulation rates in the early Caenorhabditis elegans embryo
Source: Sci Adv. 2023 Aug 23;9(34):eadi1270. doi: 10.1126/sciadv.adi1270 (PMC10446496; doi:10.1126/sciadv.adi1270)
Supplement: Supplementary file 1 — Figs. S1 to S8 Table S1 Legend for data file S1 [file sciadv.adi1270_sm.pdf]

Science Advances
AAAS

Supplementary Materials for

Transcript accumulation rates in the early *Caenorhabditis* elegans embryo

Priya Sivaramakrishnan *et al.*

Corresponding author: John Isaac Murray, jmurr@pennmedicine.upenn.edu

Sci. Adv. **9**, eadi1270 (2023)
DOI: 10.1126/sciadv.adi1270

The PDF file includes:

Figs. S1 to S8
Table S1
Legend for data file S1

Other Supplementary Material for this manuscript includes the following:

Data file S1

Supplementary Figures and Legends

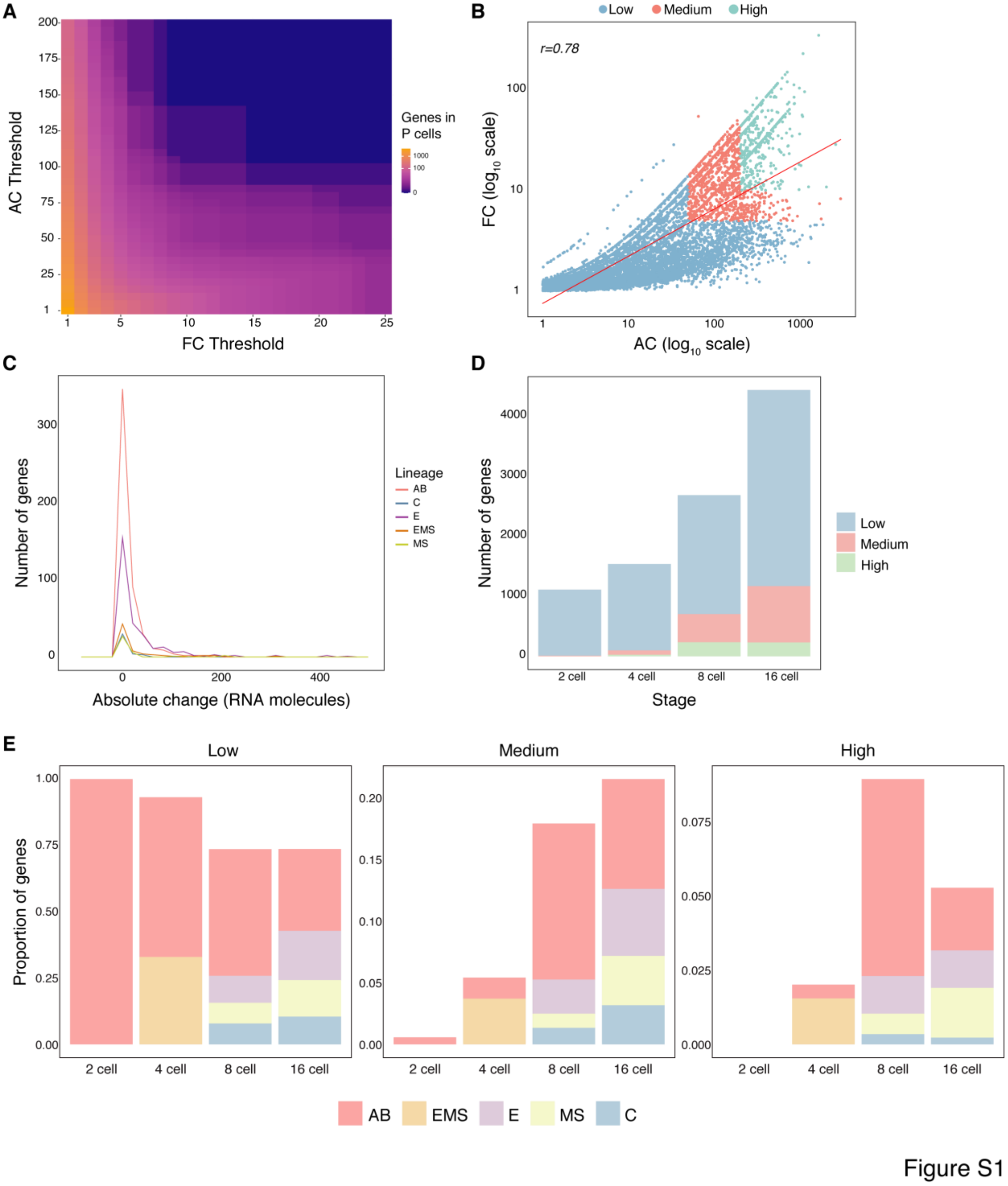

Figure S1 - Related to Fig. 1: Use of absolute change and fold change to categorize accumulation rates of genes. A. Heatmap of all absolute change (AC) and fold change (FC) as related to the number of genes at each combination of AC and FC in the germline (P cells). **B.** Linear correlation between AC and FC across different rate categories

(scale is log_{10} for visualization). **C.** Histogram distribution of positive (AC >1, FC > 1) unique to the indicated founder lineages. **D.** Number of genes in each rate category at each embryonic cell stage (related to Fig. 1C). **E.** Proportion of genes in each rate category at different embryo stages and indicated lineages.

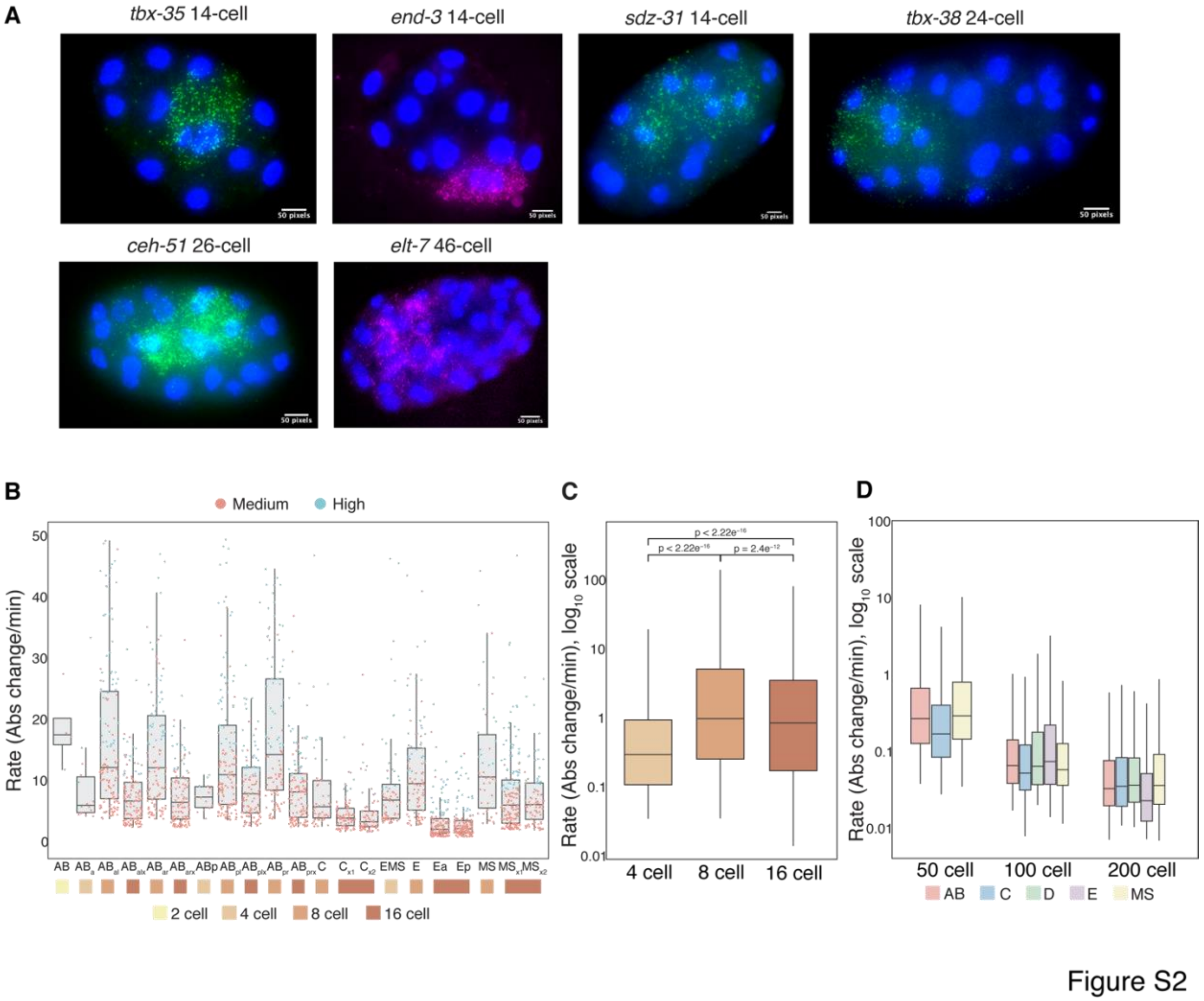

Figure S2 - Related to Fig. 2: Rates estimated from scRNA-seq across cell types and lineages.

A. Representative smFISH images at stages of maximal expression for genes in Figs. 2C-H. **B, C.** Accumulation rate breakdown for high and medium categories by cell type and embryo stage. *p*-values from Wilcoxon test in C. **D.** Rate breakdown by lineage from the Packer et al. (*30*) data for later embryo stages.

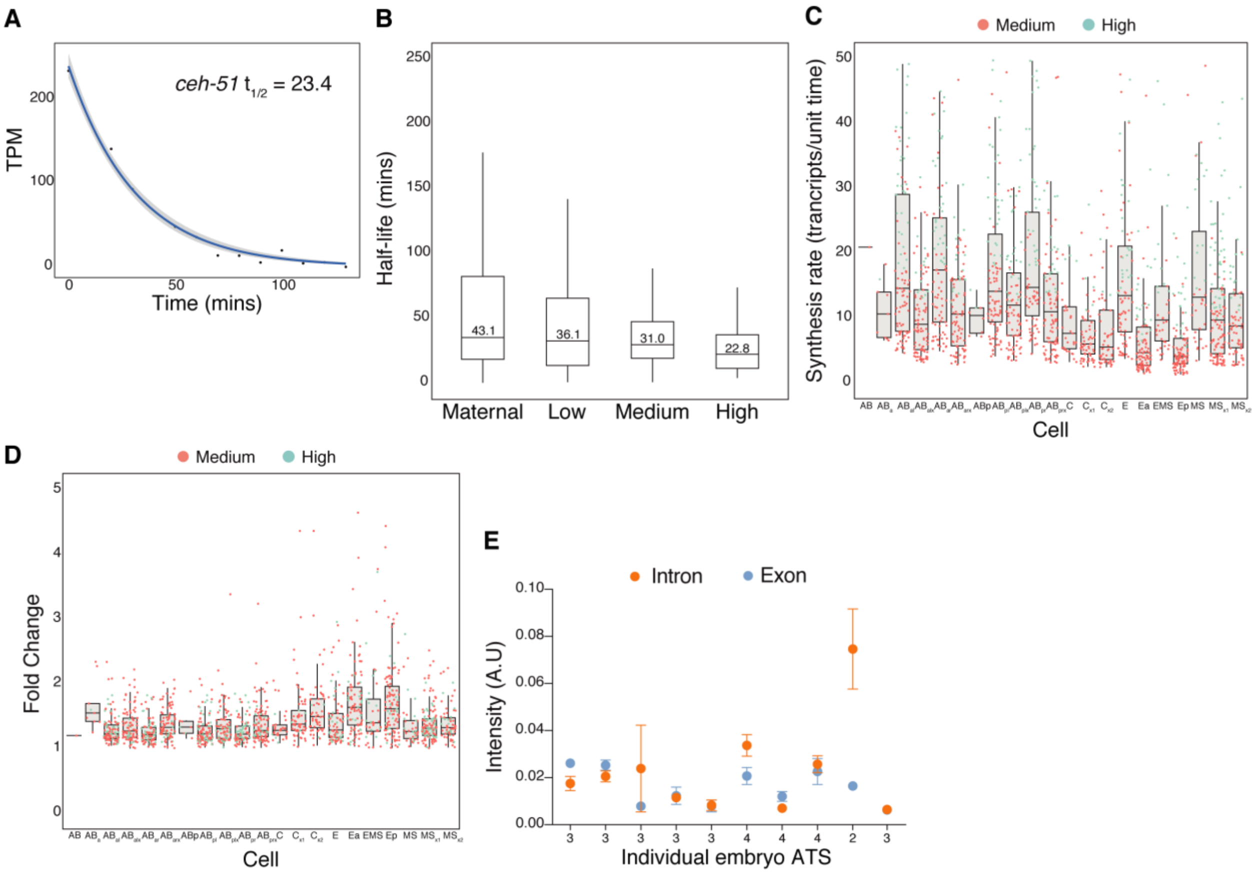

Figure S3

Figure S3 - Related to Fig. 3: Half-life estimates and adjusted synthesis rates.

A. Example for half-life estimation using one-phase decay for *ceh-51* RNA using embryo time course data (*37*). **B.** Half-lives by accumulation rate categories, median indicated for each category. **C.** Adjusted synthesis rate for medium- and high-rate genes using estimated half-lives by cell type (compare to Fig. S2B). **D.** Fold difference between accumulation rate and adjusted synthesis rate for high- and medium-rate genes across cells. **E.** Intensities of *ceh-51* exon and intron smFISH spots for individual 14- or 15- cell stage embryos. Intensities are an average +/- SEM of the number of ATS (x-axis) for each embryo.

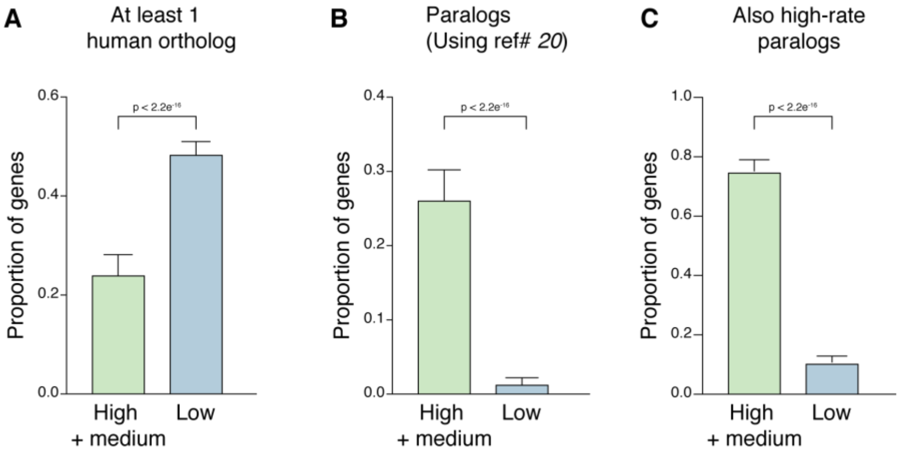

Figure S4

Figure S4 – Related to Fig. 4: Paralogs or orthologs in low *vs.* high-rate genes.

A. Proportion of all high- and low-rate genes with at least one human ortholog using Ortholist 2 (*43*). **B.** Paralogs (Blast result from a e-value threshold of 10^{-15}) that are also syn-expressed from Tintori et al. (*20*). **C.** Proportion of all high-rate *vs.* all genes that have paralogs that are also high-rate. *p*-values from Fisher's Exact Test.

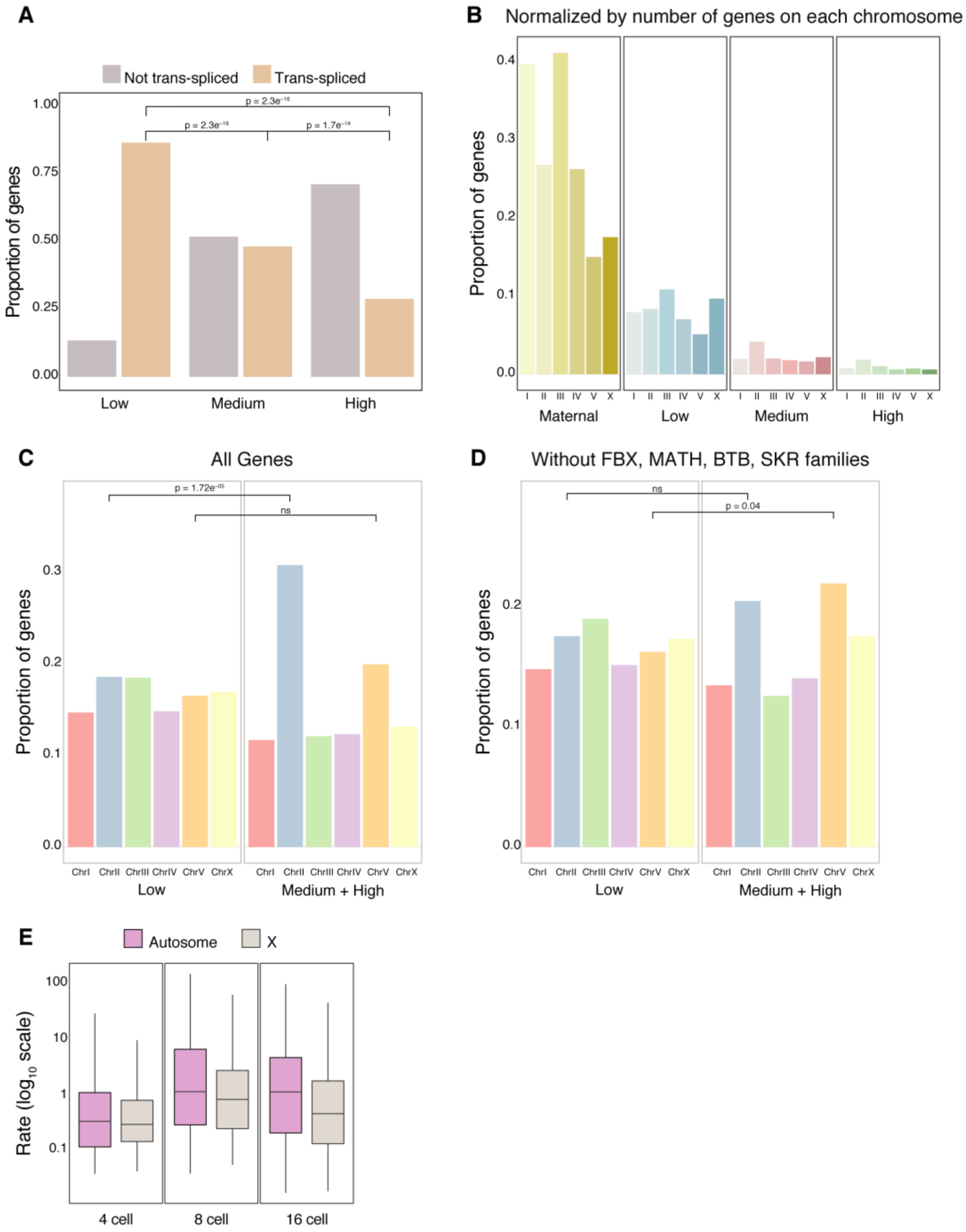

Figure S5

Figure S5 – Related to Fig. 5: Trans-splicing and distribution of genes on the different chromosomes. A. Proportion of genes that are trans-spliced or not in each rate group using data from Saito et al. (*49*). Wilcoxon Test with adjusted *p*-values. **B.** Proportion of genes in each rate category normalized by the number of genes on each chromosome. **C.** Number of genes as a proportion of the genes in either low- or medium+high- rate categories found

on the different chromosomes in all *vs.* **D.** after removing genes belonging to the FBOX, MATH, BTB and SKR families. *p*-values from Fisher's exact test. **E.** Accumulation rates by stage and type of chromosome (X *vs.* autosome).

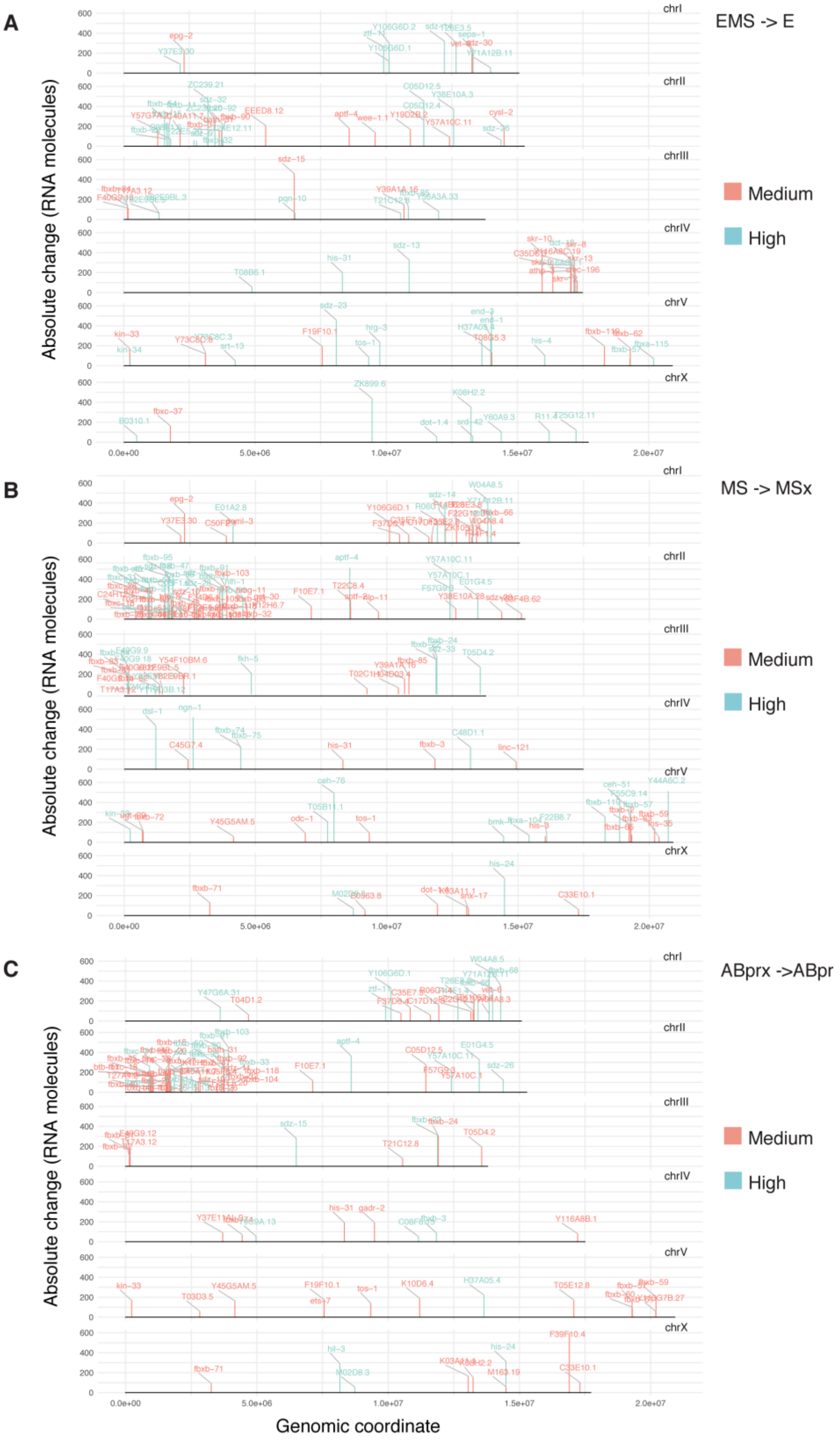
A
EMS -> E
B
MS -> MSx
C
ABprx ->ABpr
Absolute change (RNA molecules)
chrI
chrII
chrIII
chrIV
chrV
chrX
Medium
High
0.0e+00
5.0e+06
1.0e+07
1.5e+07
2.0e+07
Genomic coordinate

Figure S6

Figure S6 – Related to Fig. 5F: Karyogram of clustered genes on all chromosomes for indicated cell types.

A. Karyogram showing absolute change of genes over genomic position (coordinate) for genes in E cells with Fold Change > 5 over the parent EMS cell. **B and C.** Same as A but for MSx1 cell compared to MS parent (B) and for ABprx compared to ABpr parent (C). Genes are colored as high- or medium rate.

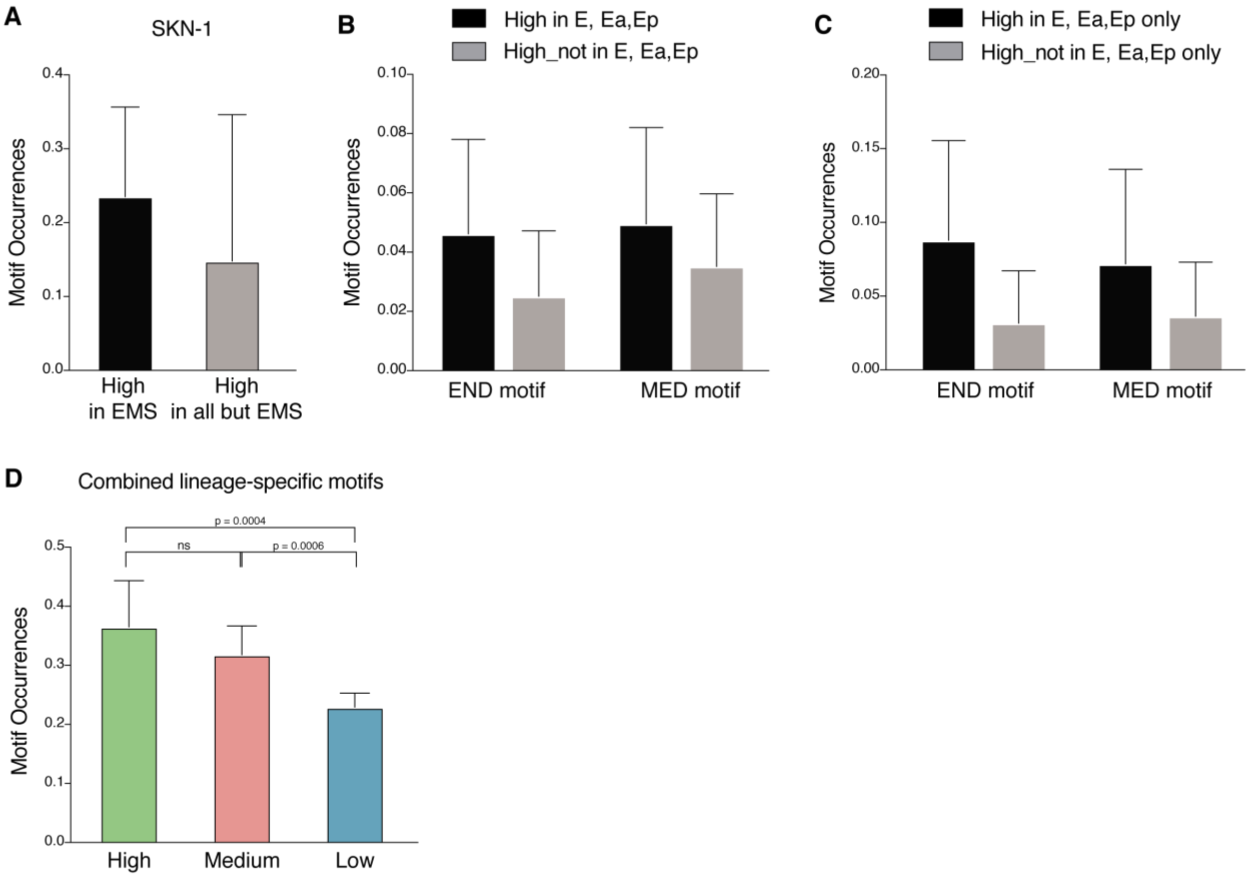

Figure S7

Figure S7 – Related to Fig. 6: Motif enrichment of lineage-specific TFs.

A. SKN-1 motif enrichment in genes expressed in the EMS compared to those expressed in all cells except EMS. **B, C.** END and MED motif enrichment in the E lineage (E, Ea, Ep cells) compared with genes expressed in all but the E lineage. C shows enrichment in genes unique to E *vs*. not. **D.** Motif occurrences for all lineage-specific motifs (in Fig. 6A) combined across the three rate categories. *p*-values from Chi-squared Test, FDR adjusted.

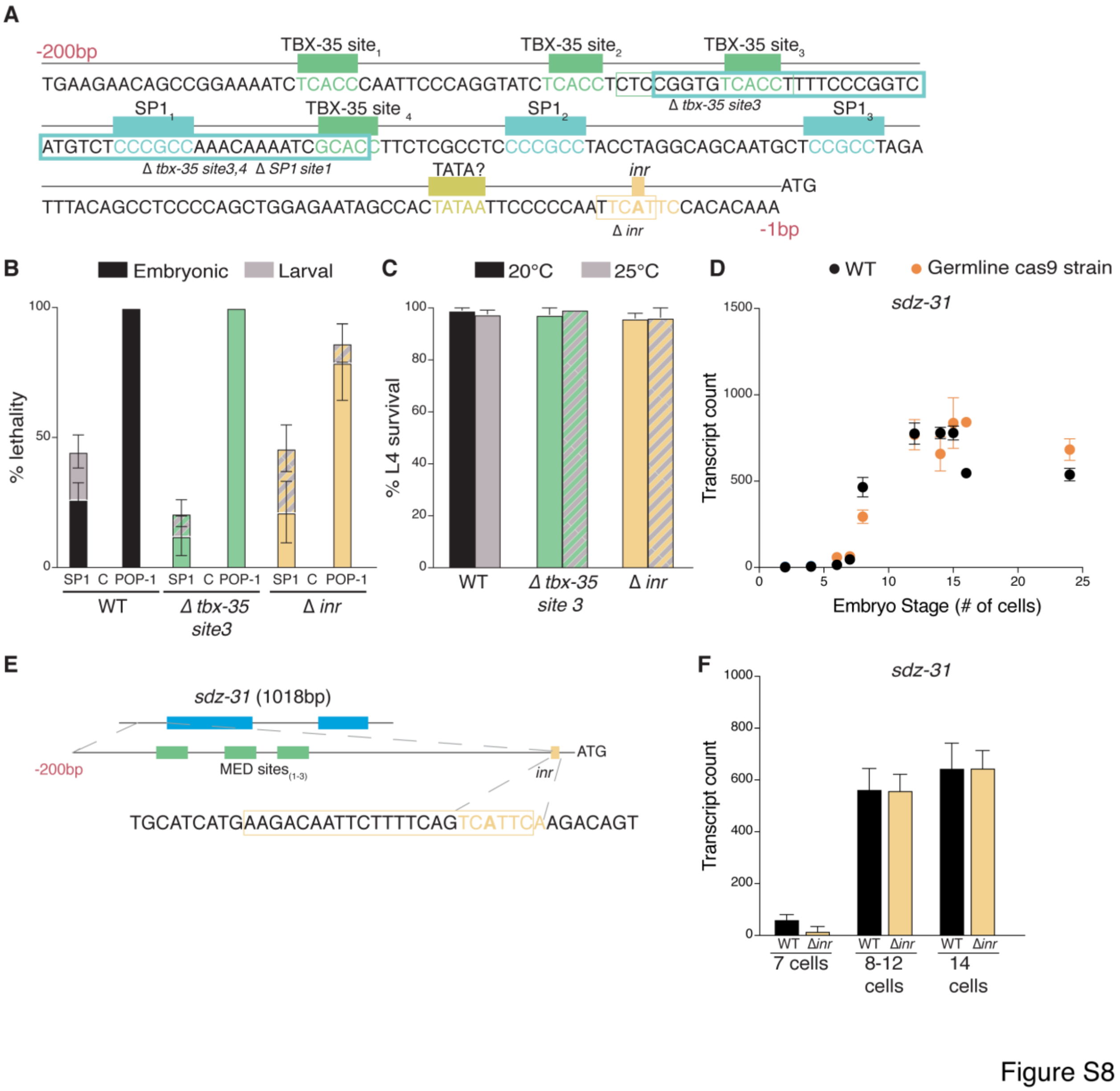

Figure S8 – Related to Fig. 7: *ceh-51* and *sdz-31* promoter region and survival of motif deletion mutants.

A. Detailed sequence of up to -200bp from the ATG start codon of the *ceh-51* promoter. Motifs indicated in filled colored boxes. Colored boxes around the sequence shows the deleted regions in indicated mutants. **B.** Embryonic and larval lethality after treatment with *sptf-3* RNAi (SP1), vector only (C) or *pop-1* RNAi, N=at least 4 biological replicates. **C.** Survival in motif mutants at 20°C and 25°C, N=at least 4 biological replicates. **D.** smFISH counts for *sdz-31* over developmental time comparing WT and the germline cas9 strain (EG9882). **E.** 200bp upstream region (from translation start ATG) of *sdz-31* showing important motifs and deletion in the Inr mutant. **F.** smFISH counts of *sdz-31* at the indicated stages in WT and the Inr promoter mutant.

High/Medium Gene Sequence Name	Public Name	WB Gene ID	DBD	Lineage specification function (incomplete) (* Suggested candidates for future studies)
C28G1.4	C28G1.4	*WBGene00016189*	ZF - C2H2 - 5 fingers	
C35D6.4	C35D6.4	*WBGene00007961*	ZF - CCCH - 2 domains	
F38C2.5	*ccch-2*	*WBGene00009537*	ZF - CCCH - 2 domains	
R13A5.5	*ceh-13*	*WBGene00000437*	HD - HOX	ABp lineage specification
Y80D3A.3	*ceh-51*	*WBGene00013583*	HD - HOX	MS lineage specification
Y116A8C.17	*dct-13*	*WBGene00013794*	ZF - CCCH - 2 domains	
ZK1193.5	*dve-1*	*WBGene00022861*	HD - 2 domains	
C18G1.2	*elt-7*	*WBGene00015981*	ZF - GATA	E lineage specification
F58E10.2	*end-1*	*WBGene00001310*	ZF - GATA	E lineage specification
F58E10.5	*end-3*	*WBGene00001311*	ZF - GATA	E lineage specification
F19F10.1	F19F10.1	*WBGene00017598*	WH - ETS	
F19F10.5	*ets-7*	*WBGene00017601*	WH - ETS	*Posterior specific-expression
F26F4.8	F26F4.8	*WBGene00005011*	ZF - C2H2 - 6 fingers	
F28C6.1	*aptf-4*	*WBGene00009202*	AP-2	
F38C2.7	F38C2.7	*WBGene00009539*	ZF - CCCH - 2 domains	
F40G9.14	F40G9.14	*WBGene00018248*	ZF - C2H2 - 1 finger	
C29F7.5	*fkh-4*	*WBGene00001436*	WH - Fork Head	
F26A1.2	*fkh-5*	*WBGene00001437*	WH - Fork Head	
F53B2.6	*ham-1*	*WBGene00001820*	WH	
B0304.1	*hlh-1*	*WBGene00001948*	bHLH	MS lineage specification
C17C3.7	*hlh-25*	*WBGene00001964*	bHLH	
C17C3.8	*hlh-26*	*WBGene00001965*	bHLH - 2 domains	ABp lineage specification
C17C3.10	*hlh-27*	*WBGene00001966*	bHLH	ABp lineage specification
T05A7.4	*hmg-11*	*WBGene00001976*	AT Hook x3	
T24D3.1	*med-1*	*WBGene00003180*	ZF - GATA	E lineage specification
K04C2.6	*med-2*	*WBGene00003181*	ZF - GATA	E lineage specification
Y69A2AR.29	*ngn-1*	*WBGene00003595*	bHLH	ABala/ABara fate specification?
T27B7.6	*nhr-228*	*WBGene00020852*	ZF - NHR	
Y22F5A.1	*nhr-232*	*WBGene00012494*	ZF - NHR	
T05B4.2	*nhr-57*	*WBGene00003647*	ZF - NHR	
C34C6.6	*prx-5*	*WBGene00004194*	Unknown	
ZK593.4	*rbr-2*	*WBGene00004319*	ARID/BRIGHT	
C47C12.3	*ref-2*	*WBGene00004335*	ZF - C2H2 - 3 fingers	Multiple anterior lineages
F12E12.5	*sdz-12*	*WBGene00017406*	ZF - C2H2 - 7 fingers	*ABal specific-expression
ZK892.7	*sdz-38*	*WBGene00014131*	ZF - C2H2 - 2 fingers	
T22C8.5	*sptf-2*	*WBGene00011926*	ZF - C2H2 - 3 fingers	
T22C8.4	T22C8.4	*WBGene00011925*	ZF - C2H2 - 1 finger	
T24C4.2	T24C4.2	*WBGene00020758*	Unknown	
F40H6.4	*tbx-11*	*WBGene00006547*	T-box	
ZK380.1	*tbx-32*	*WBGene00006551*	T-box	ABa lineage specification
ZK177.10	*tbx-35*	*WBGene00006554*	T-box	MS lineage specification
Y47D3A.12	*tbx-37*	*WBGene00006556*	T-box	ABa lineage specification
C24H11.3	*tbx-38*	*WBGene00006557*	T-box	ABa lineage specification
Y73F8A.16	*tbx-39*	*WBGene00006558*	T-box	ABa lineage specification
Y73F8A.17	*tbx-40*	*WBGene00006559*	T-box	ABa lineage specification
Y116A8C.19	Y116A8C.19	*WBGene00013796*	ZF - CCCH - 2 domains	
Y116A8C.20	Y116A8C.20	*WBGene00013797*	ZF - CCCH - 2 domains	
Y116A8C.22	*athp-3*	*WBGene00013799*	AT Hook x5	
Y60A9.3	Y60A9.3	*WBGene00013370*	ZF - CCCH - 2 domains	
Y82E9BR.1	Y82E9BR.1	*WBGene00022334*	ZF - C2H2 - 2 fingers	
Y95B8A.8	Y95B8A.8	*WBGene00022388*	ZF - C2H2	
F52F12.6	*ztf-11*	*WBGene00009939*	ZF - C2HC - 2 fingers	
Y66D12A.12	*ztf-29*	*WBGene00013438*	ZF - C2H2 - 2 fingers	

Supplementary Table 1. High and medium-rate genes annotated as transcription factors in the wTF3.0 transcription factor database.

Auxiliary Excel File - Source Data File.xlsx
